# Local autologous blood and corticosteroid injection on pain and function in patients with tennis elbow

**DOI:** 10.22088/cjim.14.4.633

**Published:** 2023

**Authors:** Rahmatollah Jokar, Seyyed Mokhtar Esmaeilnejadganji, Ali Bijani, Sekineh Kamali Ahangar, Raheleh Javer, Ghazal Mohammadi

**Affiliations:** 1 Clinical Research Development Center, Shahid Beheshti Hospital, Babol University of Medical Sciences, Babol, Iran; 2 Department of Orthopedic Surgery, School of Medicine, Babol University of Medical Sciences, Babol, Iran; 3 Social Determinants of Health Research Center Health Research Institute Babol University of Medical Sciences; 4 Student Research Committee, Babol University of Medical Sciences, Babol, Iran

**Keywords:** Tennis elbow, Lateral epicondylitis, autologous blood, Corticosteroid, Brace.

## Abstract

**Background:**

Tennis elbow or lateral epicondylitis is a common complaint involving about a 3-5% cases in a community. Non-surgical treatment is effective in 80% of cases. Recent studies have shown the effect of autologous blood on improving the pain and function of affected patients. The present study aimed to compare the effectiveness of steroid and autologous blood local injection in controlling pain and disability in the short and long term.

**Methods:**

The present study was a clinical trial conducted in Shahid Beheshti Hospital of Babol. A total of 60 patients were divided into 3 groups; A group injected at the site of lateral epicondylitis with steroid (methylprednisolone acetate-40mg) and another group with autologous blood (2ml of venous blood), and the other group used a brace for 3 weeks. Patients were followed-up for 15, 30, and 90 days, and the PRTEE assessment questionnaire assessed their pain and disability.

**Results:**

On the 15th day, there was no statistically significant difference in pain and function in the three groups, although the injectable groups were relatively more effective. On the 30th day (p=0.001), the local corticosteroid was significantly better than the autologous blood group, while on the 90th day (p<0.001), autologous blood was significantly better than the local corticosteroid. The average day, in which 25% improvement was gained, was lower in the autologous blood transfusion group.

**Conclusion:**

Regarding the long-term effect of autologous blood on corticosteroid injections, it was recommended as a lateral epicondylitis treatment.

Lateral epicondylitis is a common elbow disorder mainly due to repeated movements of wrists and fingers and stress in the origins of extensor muscles (1). The incidence of lateral epicondylitis has been reported to be 1% to 3% in various studies. Lateral epicondylitis can cause pain and dysfunction of patients in many daily activities such as carrying things, and it involves 4-7% of general population and 12.3-17.2% of workers in harmful occupations (2). Although “epicondylitis” and “tendinitis” are generally used to describe tennis elbow, histopathological studies have indicated that tennis elbow is not an inflammatory state. However, a fibroelastic and vascular response called angiofibroblastic degeneration is more well-known than tendinosis (3). Treatment of this disease is common in both surgical and non-surgical procedures. Many non-surgical treatments have been used to treat tennis elbows with unpredictable effects. Among non-surgical treatments, one or more combined treatments include rest, nonsteroidal anti-inflammatory drugs, brace, physiotherapy, iontophoresis, shock therapy, Botulinum toxin (BTX), corticosteroid injection, and the whole blood injection (4). 

Most non-surgical cases are supposed to suppress inflammation that does not occur in the tendinosis. Oral and injectable steroids and ice massage are signs of this approach (5). A better result was obtained in the past when a minor trauma such as a closed manipulation was done with excessive force or subcutaneous release with a scalpel or needle in a tendinosis site in lateral epicondylitis. Chemical modifiers of cell activities are carried in the blood and are called mitomorphogenic modifiers. Mitogens like the PDGF (platelet-derived growth factor) cause fibroblast mitosis and produce chemotactic polypeptides like TGF. TGF causes the migration of fibroblasts that play roles in the repair. Autologous blood injection may provide hormonal and cellular mediators that play roles in the repair chain. Therefore, the treatment of chronic tennis elbow cases was done by autologous blood injection in the lateral epicondylitis in 2003 and reported good results (6). 

An evaluation criterion was introduced in 2005 as the patient-rated tennis elbow assessment (PRTEE) that was responded to by patients or residents (7). Therefore, we sought to compare the local injectable effect of autologous blood with corticosteroids on the pain and function of tennis elbow patients referred to Shahid Beheshti Hospital of Babol during 2017-2018. 

## Methods

In the present clinical trial study, patients with lateral epicondylitis referring to Shahid Beheshti Hospital of Babol during 2017-2018 were considered the statistical population. Using studies and confirmation of statistical counseling and according to the probability of 25% drop in each group, the sample size was supposed to be 20 and 60 subjects. The simple sampling method was performed. Inclusion criteria: Patients with tennis elbow referring to Shahid Beheshti Hospital. Exclusion criteria: Patients with a history of long-term corticosteroid consumption (more than three consecutive months), rheumatoid arthritis, bilateral symptoms and deformity of elbow, patients with a history of previous injection, trauma and elbow fractures, and elbow surgery.

 Patients were randomly divided into three groups (autologous blood injection, corticosteroid injection, and brace group). Two milliliters of autologous blood of the first group were taken from the upper limb veins, and it is then mixed with 1 ml of 2% lidocaine solution and injected into lateral epicondylitis site under the Extensor Carpi Radialis Longus. In the second group, 40 mg of methylprednisolone acetate was mixed with 1 ml of 2% lidocaine and injected into the same place with the first group in the Extensor Carpi Radialis Brevis Longus. In the third group (control group), the tennis ball was alone used for 3 weeks after starting the treatment. All three groups of patients learned how to use appropriate tools and hand positions, and tennis elbow braces were given to patients in the first three weeks. All participants were evaluated on day zero (before the intervention), 15, 30, and 90. Evaluation and follow-up were done by Patient-Rated Tennis Elbow Assessment (PRTEE) questionnaires responded to patients. PRTEE questionnaire included two series of questions about patient pain and functions. Five pain questions scored from pain with zero scores to the worst possible pain with a score of ten. There were ten function questions with a score of zero for activity without any problem in ten for inability to work. The total pain and function scores were separately examined in three groups, and then their summed scores were considered as patients’ total scores. Data analysis was done using SPSSV22 by ANOVA, chi-square test, and analysis methods such as Kaplan–Meier method, log-rank test, and Cox-Regression with a confidence level and significance of p<0.05. (IRCT20180422039382N2 / IR.MUBABOL.HRI.REC.1397.074)

## Results

In the present study, 60 patients were divided into three treatment groups. A group was braced for only three weeks, another group was braced for three weeks and received corticosteroid injection, and the other group was braced and received an autologous blood injection. Among the patients who have received blood injections, 4 patients (2 patients due to increased pain and corticosteroid injections) stopped the follow-up. Among the second group of patients treated with corticosteroids, three patients discontinued follow-up (2 patients due to relapses and corticosteroid re-injection). Among patients, who only used braces, 7 ones continued follow-up (5 patients due to relapses of pain and function and the use of other therapies). Therefore, 17 patients in the corticosteroid group, 16 in the blood injection group, and 13 in the brace group completed the research. 

**Figure 1:**
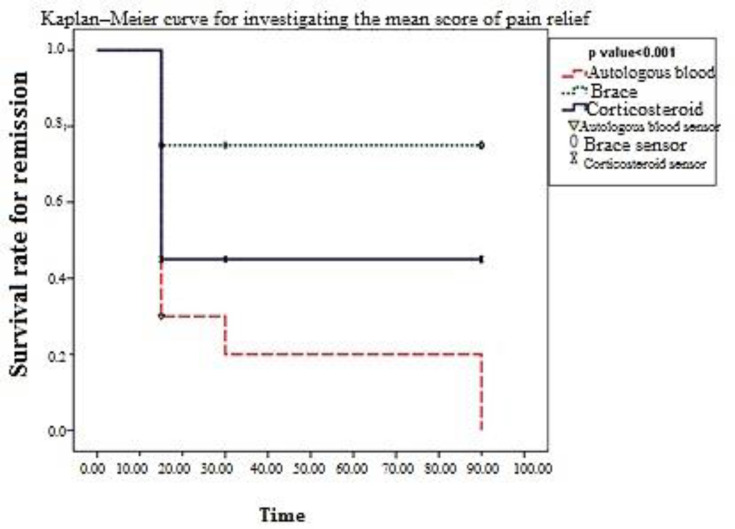
Kaplan–Meier curve for investigating the mean score of pain relief

In the present study, 34 (56.6%) women and 26 (43.3%) men participated with a women/men ratio of 1.3:1. The patients’ age range was 24-64 years. Twenty-one (45%) men and 25 (55%) women completed the study. Two cases of ecchymosis were seen in patients with corticosteroid injections and 3 cases of ecchymosis in patients with autologous blood injections. According to figure 1, the incidence of lateral epicondylitis at the age of 40 was much than those under the age of 40. 

Statistical ANOVA was used to examine and compare the impact of three therapies on the patient’s pain, and three therapeutic methods were compared. Based on the analysis of variance of patients’ pain scores, it was concluded that three therapeutic methods were not significantly different according to p-values during the treatment days of 0 and 15, but effects of treatments were significantly different on days 30-90 of follow-up. It was more effective in corticosteroids than other treatments, and patients had lower mean pain scores. However, the treatment with autologous blood was more effective, and patients had lower mean pain scores on day 90. 

Figure 1 shows the effect of different therapeutic methods on pain relief in patients. As shown, the treatment method of autologous blood injection had the greatest effect on patients’ pain relief during the treatment. Corticosteroid injection was ranked second in terms of impact, and the bracing method had the least effect on relieving the patients’ pain during the treatment. The statistical ANOVA was used to examine and compare the impact of therapeutic methods on patients’ function, and all three therapies were compared. Based on the ANOVA on patients’ function scores, there was no significant difference between therapeutic methods according to p-values on days 0 and 15 of treatment. However, the impact of methods had a statistically significant difference on days 30 and 90 of follow-up. On day 30, the corticosteroid therapy was more effective, and patients had higher mean scores than other treatments, while on day 90, the treatment with autologous blood was more effective, and patients had higher mean scores. 

**Figure 2:**
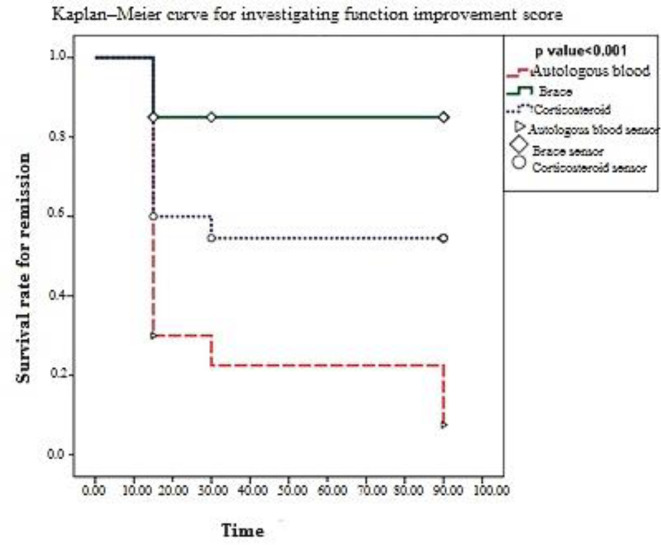
Kaplan–Meier curve for investigating the mean score of function scores

Figure 2 shows the effects of different treatment methods on the patient’s function scores. As shown, the autologous blood injection treatment method had the greatest effect on improving the patient’s function during the treatment. The corticosteroid injection method was ranked second, and the bracing method had the least effect on increasing the patient function during the treatment. 

Table1 analyzes patients’ total scores in the treatment process. There were significant differences between the three therapeutic methods according to the p-values on treatment days 0 and 15, but no significant difference between follow-up methods on days 30 and 90. 

Kaplan–Meier method was used to measure the survival rate for remission, considering the remission rate of 25%. According to the studied meantime of painful recovery, Kaplan–Meier method and log-rank test indicated that the treatment with autologous blood reduced pain by 25% on 31.5 ± 7.7 days with a confidence interval of 16.41-46.58 days. In addition, the mean pain relief in brace treatment was 71.25 ± 7.26 days with a confidence interval of 57.02 to 85.48 days. In the corticosteroid-treated group, the mean day of pain relief was 8.34 ± 48.75 days with a confidence interval of 32.40 to 65.10 days, and results of this test indicated significant differences between the three groups in terms of mean day of pain relief. (p <0.001) 

According to Kaplan–Meier method, for investigating the mean day of function relief, the treatment with autologous blood reduced the function score by 25% on day 33.00± 7.5 with a confidence interval of 18.29 to 47.71 days. In addition, it was 78.75 ± 5.90 days with a confidence interval of 67.01 to 90.49 days for evaluating the mean day of improvement by bracing. In the corticosteroid-treated group, the mean recovery day was 8.23± 56.73 with a confidence interval of 72.86 to 40.59. The results indicated significant differences between the three groups regarding improved function (p <0.001). Results in table2 indicate that the autologous blood injection treatment method is more effective than the other two methods concerning age and sex in reducing pain and improving the patients’ function by 25%.

**Table 1:** Comparison of Index scores in three groups at separate times

**Index**	**Time**	**Type of intervention**			**P-value**
		**Brace** **(mean±SD)**	**Corticosteroids** **(mean±SD)**	**Autologous blood** **(mean±SD)**	
**pain score**	Before treatment	20.80±5.69	21.65±5.26	21.80±5.85	0.832
	Day 15	14.83±5.01	13.00±3.85	13.87±4.28	0.832
	Day 30	17.13±5.74	10.72±3.89	13.50±3.76	0.001*
	Day 90	20.61±6.66	13.82±5.94	10.00±3.26	<0.001*
**function scores**	Before treatment	51.95±12.52	55.45±11.69	55.10±13.96	0.636
	Day 15	40.17±11.16	35.21±9.17	37.84±11.97	0.385
	Day 30	43.40±12.92	29.89±7.74	36.06±8.18	0.001*
	Day 90	49.85±12.67	38.82±13.10	27.81±8.28	<0.001*
**total scores**	Before treatment	72.05±17.06	77.10±16.73	76.70±19.54	0.611
	Day 15	55.89±17.26	49.89±13.77	53.26±18.05	0.543
	Day 30	60.53±18.41	40.44±11.78	51.87±15.05	0.002*
	Day 90	69.54±18.45	51.00±18.12	37.81±11.22	<0.001*

* Significant by ANOVA test.

**Table 2:** Mean days of pain relief and improved function in different treatment groups

**Index**	**Group**	**recovery day (mean±SD)**	**Confidence interval of 95%**	**P-value**
**pain relief**	Autologous blood	31.5±7.7	46.58-16.41	<0.001*
	Corticosteroid	48.75±8.34	65.10-32.40	
	Brace	71.25±7.26	85.48-57.02	
**improved function**	Autologous blood	33.00±7.5	47.71-18.29	<0.001*
	Corticosteroid	56.73±8.23	72.86-40.59	
	Brace	78.75±5.90	90.49-67.01	

* Significant by Kaplan–Meier method and Log-Rank test.

## Discussion

In the present study, 60 patients with lateral epicondylitis were evaluated, of which 26 (43.3%) were males, and 34 (56.7%) were females, with a sex ratio of 1.3:1. The mean age of patients was 48.18 years, with the range of 24-66 years. 

A topical corticosteroid is a common approach to the treatment of musculoskeletal disorders. This is a simple and inexpensive way to reduce pain and other symptoms associated with inflammation. Several biological hypotheses have been proposed to approve the effects of corticosteroids on pain control, such as prostaglandin suppression, connective tissue modification, extracellular matrix, adjustment of pain receptors and chemical intermediates, and correction of relationships between tendon structures and para-tendinosis tissues (8). 

Despite the non-approval of the hypotheses mentioned above, the effectiveness of corticosteroids is clinically shown in pain relief. In fact, the evidence suggests that corticosteroids are more useful than other treatments for short-term pain relief (9). However, no data support the effects of corticosteroids in the long term. We found no side effects in the two treatment groups. However, the repeated injection of topical corticosteroid has been reported to have adverse effects of rupture or weakness of Extensor Carpi Radialis Longus, infection, subcutaneous fat tissue atrophy, and hypopigmentation of the skin. 

In the past, it was found that the patient status was recovered when the minor trauma emerged on the lateral epicondylitis site. After releasing the tendon insertion in lateral epicondylitis, Wadsworth found good results by manipulating the package under anesthesia (10). Baumgard and Schwartz reported excellent results in 32 out of 35 patients by releasing the percutaneous tendon of Extensor Carpi Radialis Brevis (11). These measures are largely abandoned due to the increased risk of harmful consequences such as fracture, neuromuscular damage, ligament fracture, or contraction. Edward suggested that the recorded benefits of these harmful techniques could be due to a degree of bleeding at the injury site (12). Recent trials have proven that using autologous blood or platelet concentrates as topical injection play an important role in treatment, probably due to the effects of growth factors on platelets. 

Our studies indicated that patients’ pain scores decreased and patients’ function scores improved in all three groups of corticosteroids, autologous blood, and brace injection on the 15th day of follow-up. According to statistical analyses, there were no significant differences between mentioned treatments, and all of them equally affected the pain relief and function improvement. Sirico et al. found a relative priority in corticosteroid to autologous blood in recording points’ VAS scores in a 2-week follow-up (13). 

On the 30th day of follow-up, decreased pain and function scores (function improvement) were seen in both groups, but this reduction was better in the corticosteroid group than the blood injection group and was statistically significant (p< 0.05). This finding was consistent with studies by Roy et al., but Kazemi et al. found the same effect on corticosteroid and autologous blood injection at four weeks of follow-up, but autologous blood seemed to be more effective (14, 15). Jan et al. and Ariket et al. found lower mean scores of VAS on the sixth week of follow-up in patients treated with methylprednisolone than those treated with autologous blood (6, 16). The difference was significant. In the fourth week of follow-up, Dojode et al. found a significant decrease in the mean score of VAS in the corticosteroid injection group compared to autologous blood; and the difference was statistically significant (17). Edward et al. reported a complete loss of pain after three weeks of autologous blood injection (12). 

On the 90th day of follow-up, there was a decrease in pain and function scores (function improvement) in both groups compared to the injection day. In the corticosteroid injection group, the mean score of PRTEE had an increase of about 3 scores compared to the follow-up day of the 30th day, indicating the relative relapse of pain in patients of this group. However, in the group with autologous blood injection, the pain relief was seen compared to the 30th day of follow-up, indicating the slower trend of autologous blood therapy, but its long-term effect was more obvious than corticosteroid. Analysis of data was statistically significant (p< 0.05). Keratakanavar et al. found significant differences between two corticosteroids and autologous blood groups on the 12th week, and both scores of VAS and the Nisrschl stage were lower in the autologous blood group (18). Kazemi et al. in 8 weeks of follow-up and Creany et al. in 28 weeks of follow-up expressed the effectiveness of autologous blood than corticosteroid injection (15, 19). 

A prospective randomized study reported a significant reduction in pain 6 weeks after topical corticosteroid injection in 58 patients with lateral epicondylitis. However, the relapse rate was higher after 3 months (20). In another study, the relapse rate was 54% in patients treated with a topical corticosteroid (21). 

In a study for determination of mean day when patients were obtained 25% of the recovery in different treatment groups, the autologous blood injection group sooner achieved 25% of recovery than the other treatment groups reflecting the more effective autologous blood injection than corticosteroid and brace. The difference was statistically significant. Arik et al. found a complete recovery rate of 95% in autologous blood injection during 3 months, while it was 62.5% in the corticosteroid group (16).

According to this study, it was concluded that the topical corticosteroid injection had a sooner analgesic effect and a greater effect than autologous blood in the short-term treatment for up to a month, but the relapse (increased pain score and loss of function) was seen in patients up to three months. The autologous blood had a slower effect than topical corticosteroid injection, but it had a lasting effect than topical corticosteroid injection and reduced the patient need for repeated injection in the long term. 
